# The Effectiveness of Serious Games in Alleviating Anxiety: Systematic Review and Meta-analysis

**DOI:** 10.2196/29137

**Published:** 2022-02-14

**Authors:** Alaa Abd-alrazaq, Mohannad Alajlani, Dari Alhuwail, Jens Schneider, Laila Akhu-Zaheya, Arfan Ahmed, Mowafa Househ

**Affiliations:** 1 Division of Information and Computing Technology, College of Science and Engineering Hamad Bin Khalifa University Qatar Foundation Doha Qatar; 2 Artificial Intelligence Center for Precision Health Weill Cornell Medicine-Qatar Qatar Foundation Doha Qatar; 3 Institute of Digital Healthcare, Warwick Manufacturing Group, University of Warwick Warwick United Kingdom; 4 Information Science Department, College of Life Sciences, Kuwait University Kuwait Kuwait; 5 Health Informatics Unit, Dasman Diabetes Institute Kuwait Kuwait; 6 Department of Adults Health Nursing, Nursing Faculty, Jordan University of Science and Technology Irbid Jordan

**Keywords:** serious games, exergames, anxiety, computerized cognitive behavioral therapy games, biofeedback games, systematic reviews, meta-analysis, mobile phone

## Abstract

**Background:**

Anxiety is a mental disorder characterized by apprehension, tension, uneasiness, and other related behavioral disturbances. One of the nonpharmacological treatments used for reducing anxiety is serious games, which are games that have a purpose other than entertainment. The effectiveness of serious games in alleviating anxiety has been investigated by several systematic reviews; however, they were limited by design and methodological weaknesses.

**Objective:**

This study aims to assess the effectiveness of serious games in alleviating anxiety by summarizing the results of previous studies and providing an up-to-date review.

**Methods:**

We conducted a systematic review of randomized controlled trials (RCTs). The following seven databases were searched: MEDLINE, CINAHL, PsycINFO, ACM Digital Library, IEEE Xplore, Scopus, and Google Scholar. We also conducted backward and forward reference list checking for the included studies and relevant reviews. Two reviewers independently carried out the study selection, data extraction, risk of bias assessment, and quality of evidence appraisal. We used a narrative and statistical approach, as appropriate, to synthesize the results of the included studies.

**Results:**

Of the 935 citations retrieved, 33 studies were included in this review. Of these, 22 RCTs were eventually included in the meta-analysis. Very low–quality evidence from 9 RCTs and 5 RCTs showed no statistically significant effect of exergames (games entailing physical exercises) on anxiety levels when compared with conventional exercises (*P*=.70) and no intervention (*P*=.27), respectively. Although 6 RCTs demonstrated a statistically and clinically significant effect of computerized cognitive behavioral therapy games on anxiety levels when compared with no intervention (*P*=.01), the quality of the evidence reported was low. Similarly, low-quality evidence from 3 RCTs showed a statistically and clinically significant effect of biofeedback games on anxiety levels when compared with conventional video games (*P*=.03).

**Conclusions:**

This review shows that exergames can be as effective as conventional exercises in alleviating anxiety; computerized cognitive behavioral therapy games and exergames can be more effective than no intervention, and biofeedback games can be more effective than conventional video games. However, our findings remain inconclusive, mainly because there was a high risk of bias in the individual studies included, the quality of meta-analyzed evidence was low, few studies were included in some meta-analyses, patients without anxiety were recruited in most studies, and purpose-shifted serious games were used in most studies. Therefore, serious games should be considered complementary to existing interventions. Researchers should use serious games that are designed specifically to alleviate depression, deliver other therapeutic modalities, and recruit a diverse population of patients with anxiety.

## Introduction

### Background

Anxiety is a normal response to situations in human life. However, excessive anxiety may be indicative of anxiety disorders, which are mental disorders characterized by apprehension, tension, uneasiness, and other related behavioral disturbances. They are potentially coupled with other physiological symptoms, such as shortness of breath, headaches, nausea, and abdominal pain [1,2]. Anxiety disorders include separation anxiety disorder, phobia, social anxiety disorder, panic disorder, and substance- or medication-induced anxiety disorder [3]. Globally, the prevalence of anxiety disorders in the general population is estimated to be 26.9% [4]. Anxiety disorders affect all age groups, including children and adolescents [5], and can be debilitating in nature, causing significant impairment in one’s social and professional functioning [6]. Evidence has revealed a strong association between anxiety and mortality rates among healthy individuals [7,8]. Anxiety contributes to a decrease in quality of life and other health-related problems [8]. Globally, over 45 million incidents are estimated to be attributed to anxiety disorders, which, in turn, are responsible for approximately 28.68 million disability-adjusted life years [9,10].

Despite the prevalence of anxiety disorders, they often go undetected and undertreated [11]. Anxiety requires treatment and management because of the stimulation of the sympathetic system, which can lead to adverse effects. Treatments for anxiety disorders can be divided into pharmacological treatments (eg, psychotropic medications) and nonpharmacological treatments (eg, cognitive behavioral therapy [CBT]) [12,13]. Although the use of pharmacological treatments can be effective for the treatment of anxiety disorders, they can cause many adverse events and would not be effective for everyone. Therefore, nonpharmacological treatments have been used to reduce anxiety levels [14,15].

One of the nonpharmacological treatments used for reducing anxiety is serious games, which are games that have a purpose other than entertainment [16-19]. In recent years, the popularity and adoption of serious games have been on the rise because of their ability to educate and influence change in one’s experience or behaviors [20,21]. Evidence suggests that serious games can enable players to experience more meaningful, engaging, and challenging learning when compared with traditional interventions or other methods used to relieve anxiety [22].

Serious games come in a variety of types and formats, such as (1) exergames, or video games that entail physical exercises (eg, fitness and balance exercises) as part of the intended gameplay; (2) computerized CBT games, which are video games that provide CBT for the users; (3) biofeedback games, which are video games that use electrical sensors attached to the participant to receive information about the participant’s body state (eg, electrocardiogram sensors) and seek to influence some of the player body’s functions (eg, heart rate); (4) attention distraction games, which are video games that are used to direct a user’s attention away from another focus or a given event; (5) brain training games, which are video games that aim to maintain or improve users’ cognitive abilities (eg, working memory, executive function, processing speed, and attention), and (6) social skills training games that use computer-based games to improve social skills and mental health.

### Research Gap and Aim

Various studies have assessed the effectiveness of serious games in alleviating anxiety. Examining and summarizing the evidence from these studies is critical to reach informed conclusions about the effectiveness of serious games in the treatment of anxiety disorders. Two published reviews summarized the evidence regarding the effectiveness of serious games on anxiety [16,17]. However, these reviews are undermined by certain shortcomings that limit the generalization of the findings. Specifically, these reviews (1) focused on only one type of serious games (ie, exergames) [16]; (2) included non-randomized controlled trials (RCTs) [16,17]; (3) focused on a specific age group (eg, adolescents) [17]; (4) did not search the main databases of the information technology and health care fields (eg, MEDLINE, PsycINFO, IEEE Xplore, and ACM Digital Library) [16,17], or (5) did not conduct meta-analyses [17]. To address the existing gaps in the literature, this review aims to assess the effectiveness of serious games in alleviating anxiety by summarizing the results of previous studies and providing an up-to-date review.

## Methods

We conducted a systematic review and meta-analysis per the PRISMA (Preferred Reporting Items for Systematic Reviews and Meta-Analyses) statement (Multimedia Appendix 1) [23]. The protocol for this review was registered at PROSPERO (International Prospective Register of Systematic Reviews; ID: CRD42021264126).

### Search Strategy

#### Search Sources

We searched the following bibliographic databases to retrieve the relevant studies: MEDLINE (via Ovid), PsycINFO (via EBSCO), CINAHL (EBSCO), IEEE Xplore, ACM Digital Library, and Scopus. These databases were searched on June 29, 2021, by the first author (A Abd-alrazaq). We also set up automatic alerts, as needed, to retrieve weekly searches for 12 weeks (ending on August 28, 2021). Furthermore, we searched *Google Scholar* to identify gray literature. We considered only the first 10 pages (ie, 100 hits), as Google Scholar retrieves a vast number of studies, and it organizes them based on their relevance. Finally, we conducted backward and forward reference list checking (ie, screening the reference lists of the included studies and relevant reviews and screening the studies that cited the included studies).

#### Search Terms

Two experts in digital mental health were consulted before developing the search query for this review, and systematic reviews of relevance to this review were checked. The search terms were chosen based on the target intervention (eg, serious games and exergames), target outcome (eg, anxiety), and target study design (eg, RCT and clinical trial). Multimedia Appendix 2 summarizes the search query used for searching each of the 8 databases.

### Study Eligibility Criteria

Only RCTs that assessed the effectiveness of serious games in alleviating anxiety levels were included in this study. Specifically, the target intervention in this review was serious games that were delivered on digital platforms such as computers, consoles (Xbox, PlayStation, etc), mobile phones, tablets, handheld devices, or any other computerized devices. Furthermore, gaming had to be an integral and primary component of the intervention. The serious games must have been used for therapeutic or preventive purposes. Nondigital games and those used for other purposes, such as monitoring, screening, and diagnosis, were excluded. RCTs on whether there were parallel RCTs, cluster RCTs, crossover RCTs, or factorial RCTs were all included, but we excluded quasi-experiments, observational studies, and reviews.

The outcome of interest in this review was *anxiety level*, regardless of the outcome measures. We included the outcome data measured immediately after the intervention rather than the follow-up data. Trials in the English language were eligible for inclusion in this review, and all other languages were excluded. Conference abstracts and posters, commentaries, preprints, proposals, and editorials were excluded. RCTs published as journal articles, conference proceedings, and dissertations were included. No restrictions related to the population, year of publication, country of publication, comparator, or study settings were applied.

### Study Selection

We identified relevant studies in the following steps. First, we exported the retrieved studies into EndNote X8 software to identify and eliminate duplicate entries. In the second step, 2 reviewers (A Abd-alrazaq and MA) independently screened the titles and abstracts of all the retrieved studies. Finally, the full texts of the studies included in the previous step were screened independently by 2 reviewers. The 2 reviewers resolved any disagreements through discussion. The interrater agreement in steps 2 and 3 were Cohen κ=0.81 and Cohen κ=0.93, respectively, indicating a perfect level of interrater agreement [24].

### Data Extraction

Two independent reviewers used Microsoft Excel to extract the data from the included studies. Multimedia Appendix 3 shows the data extraction form that was used by the 2 reviewers to extract the data precisely and systematically from the included studies. We pilot-tested the form using the 5 included studies before proceeding. Disagreements between the reviewers were resolved via discussion. We observed an interrater agreement of 0.86, indicating a perfect level of agreement [24]. Where outcome data such as mean, SD, and sample size were unavailable, we contacted the corresponding authors in an attempt to retrieve them. In this way, we managed to retrieve such information for an additional 5 studies.

### Risk of Bias Appraisal

As recommended by the Cochrane Collaboration [25], the risk of bias was assessed by 2 independent reviewers using the Risk-of-Bias 2 (RoB 2) tool. This tool appraises the risk of bias in five domains in RCTs: randomization process, deviations from intended interventions, missing outcome data, measurement of the outcome, and selection of the reported result [25]. The risk of bias judgments in these domains was used to determine the overall risk of bias for each included study. Disagreements in judgments between the 2 reviewers were resolved via discussion. Interrater agreement between the reviewers was perfect (Cohen κ=0.86) [24].

### Data Synthesis

We used a narrative and statistical approach to synthesize the extracted data. Specifically, in our narrative synthesis, we describe the characteristics of the included studies, population, intervention, comparator, and outcome measures using texts and tables. The findings of the included studies were summarized and grouped according to the type of serious games (eg, exergames, computerized CBT games, and biofeedback games). We also conducted a meta-analysis, where at least 3 studies of the same type of serious games reported sufficient data (ie, mean, SD, and number of participants in each intervention group).

We used Review Manager (RevMan 5.4; The Cochrane Collaboration) to conduct the meta-analyses. The effect of each study and the overall effect was assessed using the standardized mean difference (SMD) because the type of data for the outcome of interest (anxiety level) was continuous, and the instruments used to evaluate the outcome were diverse among the included trials. We selected the random-effects model for the analysis because of the high clinical heterogeneity between the meta-analyzed studies in terms of serious game characteristics (eg, type, duration, frequency, and period), population characteristics (eg, sample size, mean age, and health condition), and outcome measures (eg, tools and follow-up period).

When the meta-analysis showed a statistically significant difference between the groups, we examined whether this difference was clinically important. We used the concept of *minimal clinically important difference* (MCID), which refers to the smallest change in a measured outcome that a patient would deem as worthwhile and substantial enough to warrant a change in a patient’s therapy. MCID boundaries were calculated as −0.5 to +0.5 times the SMD of the meta-analyzed studies.

We calculated *I*^2^ and a chi-square *P* value to examine the degree and statistical significance of heterogeneity, respectively, in the meta-analyzed studies. A chi-square *P* value of ≤.05 suggests heterogeneous meta-analyzed studies [26]. When *I*^2^ ranged from 0% to 40%, 30% to 60%, 50% to 90%, and 75% to 100%, the degree of heterogeneity was judged as insignificant, moderate, substantial, or considerable, respectively [26].

We used the Grading of Recommendations Assessment, Development, and Evaluation (GRADE) approach to assess the overall quality of evidence resulting from meta-analyses [27]. The GRADE approach appraises the quality of evidence based on five domains: risk of bias, inconsistency (ie, heterogeneity), indirectness, imprecision, and publication bias [27]. The overall quality of the meta-analyzed evidence was appraised separately by 2 reviewers, and any differences in decisions were addressed by discussion. The reviewers’ interrater agreement was deemed perfect (Cohen κ=0.96) [24].

## Results

### Search Results

**Figure 1:**
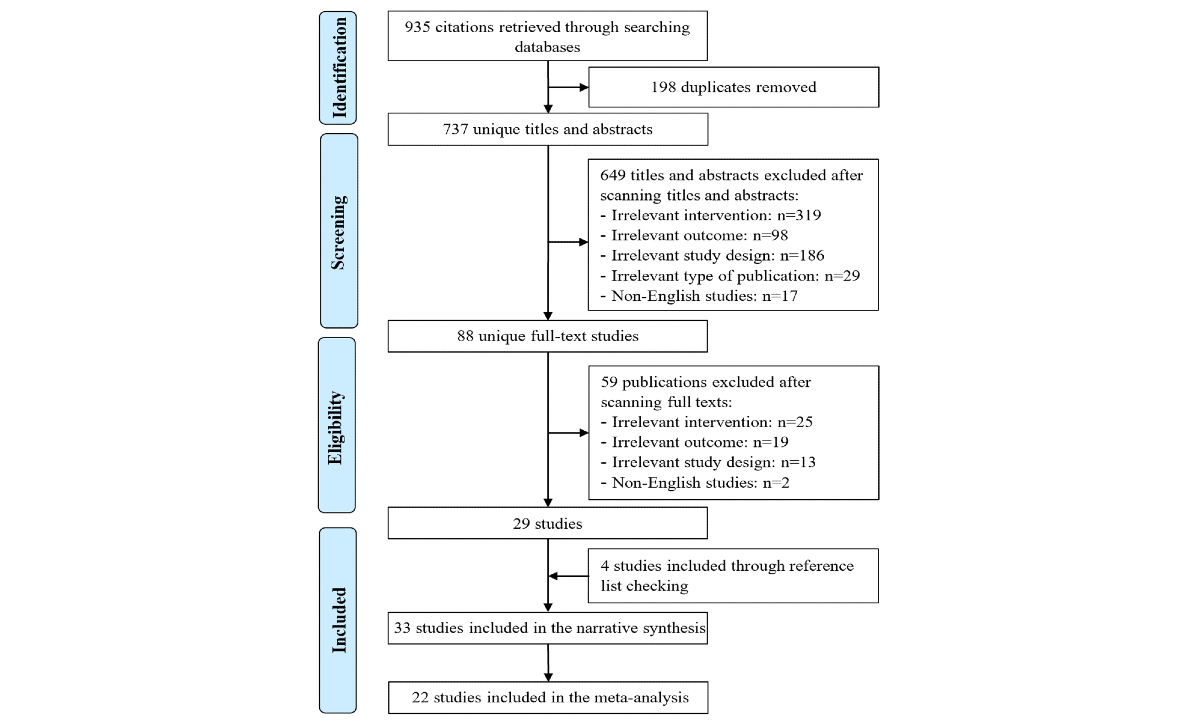
Flowchart of the study selection process.

As shown in Figure 1, we identified 935 records by searching 7 electronic databases. Of these records, we identified and removed 198 duplicates using EndNote software. The screening of the titles and abstracts of the remaining 737 records led to the exclusion of 649 citations because (1) they did not use serious games (n=319); (2) the anxiety level was not a measured outcome (n=98); (3) they were not RCTs (n=186); (4) they were not peer-reviewed articles, theses, or conference proceedings (n=29); and (5) they were published in languages other than English (n=17). Reading the full text of the remaining 88 publications led to the exclusion of 59 publications for the following reasons: (1) the intervention did not use serious games (n=25), (2) the anxiety level was not a measured outcome (n=19), (3) they were not RCTs (n=13), and (4) they were published in a language other than English (n=2). We identified 4 additional RCTs through backward and forward reference list checking. A total of 33 RCTs were included in this review [28-60]. We conducted a meta-analysis when at least 3 studies of the same type of serious games reported sufficient data (ie, mean, SD, and number of participants in each intervention group). Therefore, 22 of the included RCTs were included in the meta-analysis [28-46,49-51].

### Characteristics of Included Reviews

The included studies were published between 2012 and 2021 (Table 1). The year that witnessed the largest number of included studies was 2017 (n=8), followed by 2020 (n=6) and 2021 (n=6). The included studies were conducted in 16 different countries, as shown in Table 1. The country that published the largest number of included studies was the United States (n=6). All included studies were published in peer-reviewed journals, except for one that was a thesis. The trial type used in most of the included studies was parallel RCTs (n=31).

**Table 1:** Characteristics of studies and populations.

Study	Year	Country	Publication type	RCT^a^ type	Sample size, n	Age (years), mean	Sex (male), n (%)	Target group or condition	Setting
Adomaviciene et al [28]	2019	Lithuania	Journal article	Parallel	60	64.6	40 (66.7)	Stroke	Clinical
Carvalho et al [29]	2020	Brazil	Journal article	Parallel	35	51.3	0 (0)	Fibromyalgia	Educational
Meldrum et al [30]	2015	Ireland	Journal article	Parallel	71	54.1	27 (38)	Unilateral peripheral vestibular loss	Clinical
Schumacher et al [31]	2018	Germany	Journal article	Parallel	42	56.3	25 (59.5)	Hematopoietic stem cell transplantation recipients	Clinical
Ruivo et al [32]	2017	Ireland	Journal article	Parallel	32	59.9	26 (81.3)	Cardiovascular diseases	Clinical, community, and educational
Mazzoleni et al [33]	2014	Italy	Journal article	Parallel	40	71.2	NR^b^	Chronic respiratory diseases	Clinical
Polat et al [34]	2021	Turkey	Journal article	Parallel	40	44.8	0 (0)	Fibromyalgia	Clinical
Lin et al [35]	2020	Taiwan	Journal article	Parallel	80	57.0	39 (48.8)	Knee osteoarthritis	Clinical
Vieira et al [36]	2017	Portugal	Journal article	Parallel	46	57.7	NR	Cardiovascular diseases	Clinical
Thomas et al [37]	2017	United Kingdom	Journal article	Parallel	30	49.3	3 (10)	Multiple sclerosis	Clinical
Wagener et al [38]	2012	United States	Journal article	Parallel	41	14.0	14 (33.3)	Obese adolescents	Clinical
Jahouh et al [39]	2021	Spain	Journal article	Parallel	80	84.2	35 (44)	Older adults	Clinical
Collado-Mateo et al [40]	2017	Spain	Journal article	Parallel	83	52.5	0 (0)	Fibromyalgia	Clinical
Cooney et al [41]	2017	Ireland	Journal article	Parallel	52	40.6	20 (38.8)	Anxiety, depression, or intellectual disability	Clinical
Donker et al [42]	2019	Netherlands	Journal article	Parallel	193	41.3	64 (33.2)	Acrophobia	Community
Fish et al [43]	2014	United States	Journal article	Parallel	59	30.0	29 (49.2)	Depression	Clinical and educational
Fleming et al [44]	2012	New Zealand	Journal article	Crossover	32	14.9	18 (56)	Depression	Educational
Merry et al [45]	2012	New Zealand	Journal article	Parallel	187	15.6	64 (34.2)	Depression	Clinical and educational
Perry et al [46]	2017	Australia	Journal article	Cluster	540	16.7	199 (36.9)	Secondary students	Educational
Schoneveld et al [47]	2018	Netherlands	Journal article	Parallel	174	10.0	71 (40.8)	Anxiety	Educational
Tsui [48]	2016	Canada	Thesis	Parallel	143	13.6	52 (36.1)	Anxiety	Clinical and educational
Schoneveld et al [49]	2016	Netherlands	Journal article	Parallel	136	10.0	61 (45.2)	Anxiety	Educational
Wijnhoven et al [50]	2020	Netherlands	Journal article	Parallel	109	11.1	84 (77.1)	Anxiety and autism spectrum disorder	Clinical and educational
Scholten et al [51]	2016	Netherlands	Journal article	Parallel	138	13.3	48 (35)	Anxiety	Educational
Marechal et al [52]	2017	France	Journal article	Parallel	118	6.8	83 (70.4)	Children undergoing general anesthesia	Clinical
Sakızcı Uyar et al [53]	2021	Turkey	Journal article	Parallel	138	6.6	61 (44)	Adenoidectomy, adenotonsillectomy, or myringotomy	Clinical
Liu et al [54]	2020	United States	Journal article	Parallel	53	12.5	37 (69.8)	Otolaryngological conditions	Clinical
Butler et al [55]	2020	Germany	Journal article	Parallel	40	33.4	40 (100)	Posttraumatic stress disorder	Clinical
Bove et al [56]	2021	United States	Journal article	Parallel	44	51.1	9 (20.5)	Multiple sclerosis	Clinical
Sanchez et al [57]	2017	United States	Journal article	Parallel	69	NR	41 (59.4)	Social skills deficits	Community and educational
Beidel et al [58]	2021	United States	Journal article	Parallel	42	9.6	16 (38.1)	Social anxiety	Community
Haberkamp et al [59]	2021	Germany	Journal article	Parallel	68	22.8	9 (13)	Arachnophobia	Educational
Litvin et al [60]	2020	United Kingdom	Journal article	Parallel	709	NR	420 (59.3)	Generally healthy employees	Company

^a^RCT: randomized controlled trial.

^b^NR: not reported.

The sample size in the included studies ranged from 30 to 709, with an average of 112.8 (SD 93.2). The targeted participants were adults (aged >18 years) in 18 studies, adolescents (aged 12-18 years) in 5 studies, children (aged 5-11 years) in 5 studies, and both children and adolescents in 3 studies. Specifically, the mean age of participants reported in 31 studies ranged between 6.6 and 84.2 years, with an average of 34.7 (SD 22.4) years. The percentage of males reported in 31 studies ranged from 0% to 100%, with an average of 43.2% (SD 23.8%). The participants’ health conditions varied between studies, and anxiety was the most common (n=7). The participants in most studies were recruited from clinical settings (n=22).

Only serious games were used as interventions in 28 of the included studies, whereas the remainder used serious games combined with other interventions (Table 2). Nintendo Wii Fit (n=5) was the most common game used in the included studies, followed by MindLight (n=4). We identified eight types of serious games based on the therapeutic modality that they deliver: exergames (n=13), computerized CBT games (n=6), biofeedback games (n=5), attention distraction games (n=3), brain training games (n=2), social skills training games (n=2), exposure therapy games (n=1), and psychoeducation games (n=1). In 20 studies, games were designed with a *serious* purpose from the beginning (designed serious games); however, in the remaining 13 studies, they were not designed as serious games from the start but rather were used for a serious purpose (purpose-shifted games). The most common platforms used for playing games were computers (n=17) and video game consoles (n=8). The duration of the games in the included studies ranged from 5 to 150 minutes, but it was ≤60 minutes in most studies (n=28). The frequency of playing the games varied between only one time throughout the study and once a day, but it ranged between once a week and 3 times a week in 24 studies. The intervention period ranged from 1 to 24 weeks, but it ranged from 1 to 10 weeks in 25 studies.

As shown in Table 3, the comparison groups received inactive interventions in 14 studies while they received active interventions in 21 studies (eg, conventional exercises, CBT programs, video games, medication, and psychotherapy). Note that the numbers do not add up because 2 studies delivered both active and inactive interventions as comparators. The duration of the active comparators ranged from 10 to 180 minutes. The frequency of the active comparators varied between only one time throughout the study and once a day, but it ranged between once a week and 3 times a week in about half of the studies (15/33, 45.5%. The period of active comparators varied between 1 week and 24 weeks. The outcome of interest (eg, anxiety level) was measured using 15 different tools, but the most common tools used by the included studies were the Spence Children’s Anxiety Scale (SCAS; n=8) and the Hospital Anxiety and Depression Scale (n=7). The outcome of interest was measured immediately after the intervention in all included studies, and the most common follow-up period was 3 months (n=10). Participant attrition was reported in 32 studies, ranging from 0 to 335.

**Table 2:** Characteristics of interventions.

Study	Intervention	Serious game name	Serious game type	Serious game genre	Platform	Duration (minute)	Frequency (time per week)	Period (week)
Adomaviciene et al [28]	Serious game	N/A^a^	Exergame	Designed	Computer, Kinect	45	Once a day	2
Carvalho et al [29]	Serious game	Wii Fit Plus	Exergame	Purpose-shifted	Wii console, balance board, Wii remote plus	60	3	7
Meldrum et al [30]	Serious game	Wii Fit Plus	Exergame	Purpose-shifted	Wii console, balance board, Frii Board	15	5	6
Schumacher et al [31]	Serious game	Wii Fit, Wii Sports	Exergame	Purpose-shifted	Wii console, balance board	30	5	2
Ruivo et al [32]	Serious game	Wii Sports	Exergame	Purpose-shifted	Wii console, Kinect	60	2	6
Mazzoleni et al [33]	Serious game + pulmonary rehabilitation program	Wii Fit Plus	Exergame	Purpose-shifted	Wii console, balance board, Wii remote plus	60	7	3
Polat et al [34]	Serious game + cycling activity	Kinect Sports (Beach Volleyball)	Exergame	Purpose-shifted	Computer, Kinect	35	3	4
Lin et al [35]	Serious game + hot packs + transcutaneous electrical nerve stimulation	Hot Plus	Exergame	Designed	Computer, sensing pad	20	3	4
Vieira et al [36]	Serious game	Kinect-RehabPlay	Exergame	Designed	Computer, Kinect	70-85	3	24
Thomas et al [37]	Serious game	Wii Fit Plus, Wii Sports, Wii Sports Resort	Exergame	Purpose-shifted	Wii console, balance board, Wii remote controls	27	2	24
Wagener et al [38]	Serious game	Dance Dance Revolution	Exergame	Purpose-shifted	Computer, sensing pad	40-75	3	10
Jahouh et al [39]	Serious game	Step, Nodding	Exergame	Purpose-shifted	Wii console	40-45	2-3	8
Collado-Mateo et al [40]	Serious game	VirtualEx-FM	Exergame	Designed	Computer, Kinect	60	2	8
Cooney et al [41]	Serious game	Pesky Gnats: The Feel Good Island	CBT^b^ game	Designed	Computer	60	1	7
Donker et al [42]	Serious game	ZeroPhobia	CBT game	Designed	Smartphone, wearables (VR^c^ goggles)	5-40	2	3
Fish et al [43]	Serious game	Bejeweled II, Peggle, Bookworm Adventures	CBT game	Purpose-shifted	Computer	30	3	4
Fleming et al [44]	Serious game	SPARX	CBT game	Designed	Computer	30	1-2	5
Merry et al [45]	Serious game	SPARX	CBT game	Designed	Computer	20-40	1-2	4-7
Perry et al [46]	Serious game	SPARX-R	CBT game	Designed	Computer	20-30	1-2	5-7
Schoneveld et al [47]	Serious game	MindLight	Biofeedback game	Designed	Computer, wearables (EEG^d^ headset)	60	1	6
Tsui [48]	Serious game	MindLight	Biofeedback game	Designed	Computer	60	2	3
Schoneveld et al [49]	Serious game	MindLight	Biofeedback game	Designed	Computer, wearables (EEG headset)	60	2	3
Wijnhoven et al [50]	Serious game	MindLight	Biofeedback game	Designed	Computer, wearable (headset)	60	1	6
Scholten et al [51]	Serious game	Dojo	Biofeedback game	Designed	Computer	60	2	3
Marechal et al [52]	Serious game	N/A	Attention distraction game	Purpose-shifted	Tablet	20	One time throughout the study	N/A
Sakızcı Uyar et al [53]	Serious game	Angry Birds, Subway Surfers, Snail Bob	Attention distraction game	Purpose-shifted	Tablet	20	One time throughout the study	N/A
Liu et al [54]	Serious game + topical analgesia	SpaceBurgers	Attention distraction game	Designed	Wearables (VR goggles), handheld controller	NR	One time throughout the study	N/A
Butler et al [55]	Serious game + eye movement desensitization and reprocessing (EMDR^e^) therapy	Tetris	Brain training game	Purpose-shifted	Nintendo DS XL console	120-150	Once a day (Tetris); 2 time a week (EMDR)	6
Bove et al [56]	Serious game	Band Togather	Brain training game	Designed	Tablet	25	5	6
Sanchez et al [57]	Serious game	Adventures	Social skills training game	Designed	Computer	25	1	9
Beidel et al [58]	Serious game	Pegasys-VR	Social skills training game (Social effectiveness therapy game)	Designed	Tablet	60-120	2	12
Haberkamp et al [59]	Serious game	Spider app	Exposure therapy game	Designed	Smartphone	12	2	1
Litvin et al [60]	Serious game	eQuoo	Psychoeducation game	Designed	Smartphone, tablet	10-15	1	5

^a^N/A: not applicable.

^b^CBT: cognitive behavioral therapy.

^c^VR: virtual reality.

^d^EEG: electroencephalography.

^e^EMDR: Eye Movement Desensitization and Reprocessing.

**Table 3:** Characteristics of comparators and outcomes.

Study	Comparator	Duration (minute)	Frequency (time per week)	Period (week)	Outcome measures	Follow-up	Attrition
Adomaviciene et al [28]	Robot-assisted trainings	45	Once a day	2	HADS^a^	Postintervention	18
Carvalho et al [29]	Conventional exercises	60	3	7	Fibromyalgia Impact Questionnaire	Postintervention	14
Meldrum et al [30]	Conventional exercises	15	5	6	HADS	Postintervention	9
Schumacher et al [31]	Conventional exercises (physiotherapy)	30	5	2	HADS	Postintervention, 30- and 100-day follow-up	11
Ruivo et al [32]	Conventional exercises	60	2	6	HADS	Postintervention, 2-month follow-up	4
Mazzoleni et al [33]	Conventional exercises (pulmonary rehabilitation program)	60	7	3	STAI^b^	Postintervention	1
Polat et al [34]	Conventional exercises + cycling activity	35	3	4	HADS	Postintervention, 1-month follow-up	6
Lin et al [35]	Conventional exercises + hot packs + transcutaneous electrical nerve stimulation	20	3	4	HADS	Midintervention, postintervention, and 1 and 3-month follow-up	1
Vieira et al [36]	Conventional exercises, control	70-85	3	24	Depression Anxiety and Stress Scale 21	Midintervention, postintervention	13
Thomas et al [37]	Control	N/A^c^	N/A	N/A	HADS	Postintervention	2
Wagener et al [38]	Control	N/A	N/A	N/A	Behavior Assessment System for Children-2	Postintervention	1
Jahouh et al [39]	Control	N/A	N/A	N/A	Goldberg Anxiety and Depression Scale	Postintervention	N/A
Collado-Mateo et al [40]	Control	N/A	N/A	N/A	Fibromyalgia Impact Questionnaire	Postintervention	7
Cooney et al [41]	Control	N/A	N/A	N/A	Glasgow Anxiety Scale for people with an Intellectual Disability	Postintervention, 3-month follow-up	3
Donker et al [42]	Control	N/A	N/A	N/A	Beck Anxiety Inventory, Acrophobia Questionnaire	Postintervention, 3-month follow-up	59
Fish et al [43]	Educational website	30	3	4	State-Trait Anxiety Inventory	Postintervention	0
Fleming et al [44]	Control	N/A	N/A	N/A	SCAS^d^	Postintervention	5
Merry et al [45]	Control	N/A	N/A	N/A	SCAS	Postintervention, 3-month follow-up	17
Perry et al [46]	Control (interactive web-based program)	20-30	1-2	5-7	SCAS	Postintervention, 6- and 18-month follow-up	134
Schoneveld et al [47]	Conventional CBT^e^	60-90	1	8	SCAS	Postintervention, 3- and 6-month follow-up	36
Tsui [48]	Conventional CBT (web-based CBT)	60	2	3	SCAS, STAI	Postintervention, 3-month follow-up	19
Schoneveld et al [49]	Video game	60	2	3	SCAS	Postintervention, 3-month follow-up	21
Wijnhoven et al [50]	Video game	60	1	6	SCAS	Postintervention, 3-month follow-up	33
Scholten et al [51]	Video game	60	2	3	SCAS	Postintervention, 3-month follow-up	9
Marechal et al [52]	Midazolam	N/A	N/A	N/A	Modified Yale Preoperative Anxiety Scale	Postintervention, 2-hour follow-up	3
Sakızcı Uyar et al [53]	Midazolam, watching an informative cartoon	N/A	One time throughout the study	N/A	Modified Yale Preoperative Anxiety Scale	Postintervention	4
Liu et al [54]	Control (topical analgesia)	N/A	N/A	N/A	Subjective Units of Distress	Postintervention	0
Butler et al [55]	Eye Movement Desensitization and Reprocessing therapy	60-90	2	6	STAI	Postintervention, 6-month follow-up	0
Bove et al [56]	Video game	25	5	6	STAI	Postintervention, 2-month follow-up	4
Sanchez et al [57]	Control	N/A	N/A	N/A	Social Anxiety Scale for Children-Revised	Postintervention	24
Beidel et al [58]	Social effectiveness therapy	60-180	2	12	SPAI-C^f^	Postintervention	4
Haberkamp et al [59]	Video game	12	2	1	Survey developed by the authors	Midintervention, Postintervention	6
Litvin et al [60]	Conventional CBT, control	10	1	5	Survey developed by the authors	Midintervention, Postintervention	355

^a^HADS: Hospital Anxiety and Depression Scale.

^b^STAI: State-Trait Anxiety Inventory.

^c^N/A: not applicable.

^d^SCAS: Spence Children’s Anxiety Scale.

^e^CBT: cognitive behavioral therapy.

^f^SPAI-C: Social Phobia and Anxiety Inventory for Children.

### Results of Risk of Bias Appraisal

**Figure 2:**
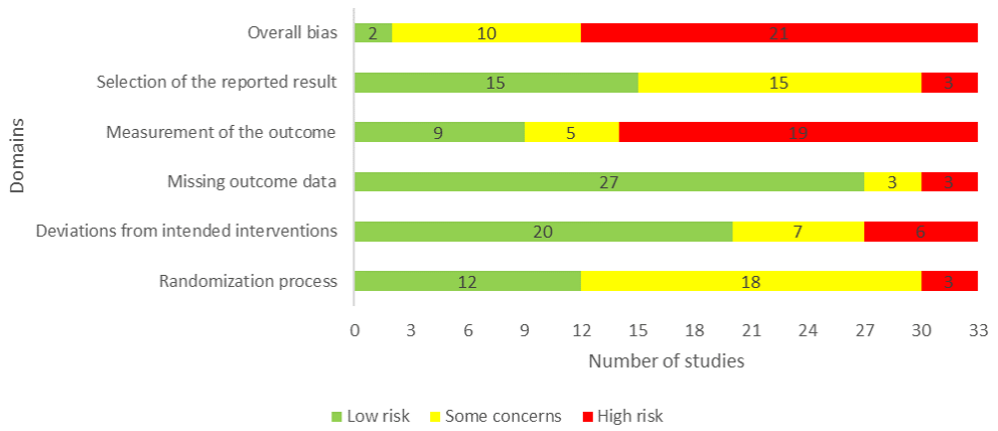
Review authors’ judgments about each risk of bias domain.

Approximately 70% (23/33) of the included studies generated an appropriate random allocation sequence for the randomization process. The allocation sequence in 14 studies was concealed until the participants were assigned to the interventions. The groups were comparable at baseline in the 29 studies. On the basis of these judgments, the risk of bias because of the randomization process was rated as low in 12 studies (Figure 2).

Participants and those who were delivering the interventions were blinded to the assigned interventions during the trial in 4 and 5 studies, respectively. In 2 studies, there was a deviation from the intended intervention, which occurred because of the experimental contexts. An appropriate analysis (eg, intention-to-treat or modified intention-to-treat analyses) was used in 26 studies to estimate the effect of the intervention. According to these judgments, the risk of bias because of deviations from the intended interventions was low in 20 studies (Figure 2).

Outcome data were available for more than 95% of the participants only in 12 studies. There was evidence that the findings were not biased by the missing outcome data in only 7 studies. In 8 studies, the missing outcome data resulted from reasons that were documented and not related to the outcome. Accordingly, 27 studies were judged as having a low risk of bias in the *missing outcome data* domain.

Four studies assessed the outcome of interest (ie, anxiety levels) using inappropriate measures. The measurement methods were comparable across the intervention groups in all included studies. The assessor of the outcome was aware of the assigned interventions in the 20 studies. Given that the outcome measure was subjective in all studies, the assessment of the outcome could have been affected by knowledge of the intervention received. Accordingly, only 9 studies were rated as having a low risk of bias in the *measuring the outcome* domain (Figure 2).

There was a prespecified analysis plan (ie, protocol) for the 15 studies. Only 3 studies reported outcome measurements that differed from those specified in the analysis plan. In all studies, there was no evidence that they selected their results from many results produced from multiple eligible analyses of the data. Accordingly, the risk of bias because of the selection of the reported results was considered low in 15 studies (Figure 2).

In the last domain, *overall bias*, the risk of bias was considered high in 21 studies as they were judged as having a high risk of bias in at least one domain. Ten studies were judged to raise some concerns in the domain of *overall bias*, as they had some concerns in at least one of the domains and were not at high risk for any domain. The 2 remaining studies were judged to be at low risk of bias for the domain of *overall bias*, given that it was rated as having a low risk of bias for all domains. Reviewers’ judgments about each *risk of bias* domain for each included study are presented in Multimedia Appendix 4 [29-60].

### Results of Studies

In this review, serious games were classified into eight types based on the therapeutic modality that they deliver: exergames [28-40], computerized CBT games [41-46], biofeedback games [47-51], attention distraction games [52-54], brain training games [55,56], social skills training games [57,58], exposure therapy games [59], and psychoeducation games [60]. The results of the included studies are shown in the following subsections based on the types of serious games.

### Exergames

#### Exergames Versus Conventional Exercises

In total, 9 studies compared the effects of exergames with conventional exercises on the level of anxiety [28-36]. While 7 studies did not find a statistically significant difference in the anxiety levels between the groups [30-36], the 2 remaining studies showed a statistically significant difference in the anxiety level between the groups (one of them favored exergames over conventional exercises [29] while the other favored conventional exercises over exergames [28]).

**Figure 3:**
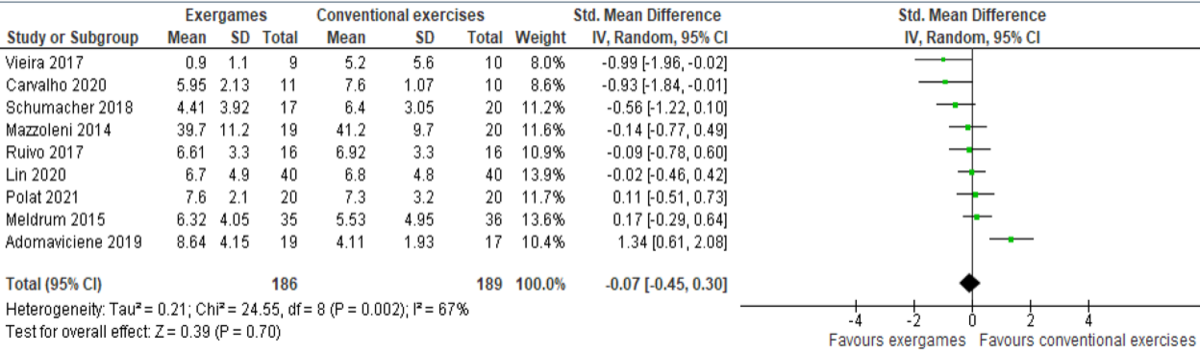
Forest plot of 9 studies comparing the effect of exergames to that of conventional exercises on the anxiety level [28-36]. Std: standardized.

The results of the 9 studies were meta-analyzed as shown in Figure 3 [28-36]. No statistically significant difference (*P*=.70) in the anxiety levels was found between the exergame group and conventional exercise group (SMD −0.07, 95% CI −0.45 to 0.30). The degree of evidence heterogeneity was substantial (*P*=.002; *I*^2^=67%). The quality of the evidence was very low, as it was downgraded by 6 levels because of a high risk of bias, heterogeneity, and imprecision (Multimedia Appendix 5).

We ran a sensitivity analysis, given that some effect sizes seem to be outliers. Specifically, we removed the study conducted by Adomaviciene et al [28] for two reasons: (1) the anxiety level at baseline was statistically higher (*P*<.001) in the exergame group (mean 9.16, SD 4.59) than in the conventional exercise group (mean 5.52, SD 2.37) and (2) the comparator was conventional exercises guided by robotic devices, which is not the case in other studies. Although the degree of heterogeneity decreased significantly from 67% to 30% by excluding Adomaviciene et al [28], there was still no statistically significant difference (*P*=.18) in the anxiety levels between groups (SMD −0.18, 95% CI −0.45 to 0.08; Multimedia Appendix 6 [28-60]).

We also reran the meta-analysis after excluding another study [36] because the anxiety level at baseline was considerably higher in the conventional exercise group (mean 8.0, SD 9.1) than in the exergame group (mean 2.7, SD 2.0). Similar to the first sensitivity analysis, the degree of heterogeneity decreased significantly from 30% to 13% by excluding Vieira et al [36]; however, there was still no statistically significant difference (*P*=.38) in the anxiety levels between groups (SMD −0.11, 95% CI −0.35 to 0.13; Multimedia Appendix 6).

#### Exergames Versus No Intervention

Five studies compared the effect of exergames with no intervention or inactive intervention on anxiety levels [36-40]. Whereas 4 studies did not find a statistically significant difference in anxiety levels between the groups [36-39], the remaining study showed a statistically significant difference in the anxiety levels between the groups, favoring exergames over no intervention [40].

**Figure 4:**
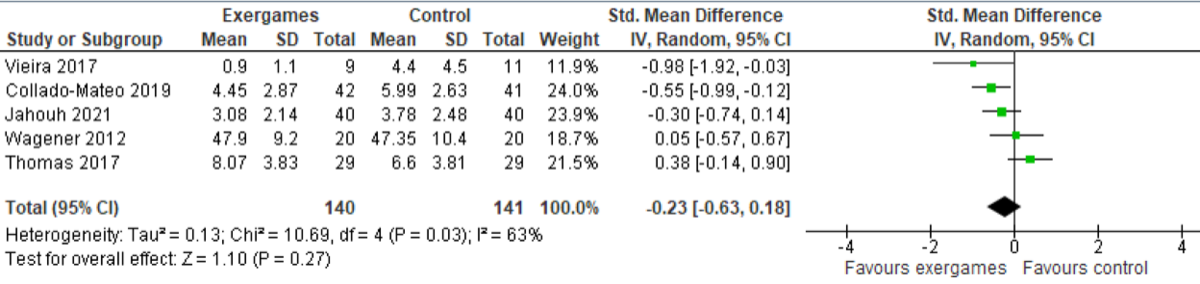
Forest plot of 5 studies comparing the effect of exergames to that of no intervention on the anxiety level [36-40]. Std: standardized.

A meta-analysis of the results of the 5 studies showed no statistically significant difference (*P*=.27) in the anxiety levels between the exergame group and the no intervention group (SMD −0.23, 95% CI −0.63 to 0.18; Figure 4 [36-40]). The heterogeneity of the meta-analyzed evidence was substantial (*P*=.03; *I*^2^=63%). The quality of the evidence was very low, as it was downgraded by 6 levels because of a high risk of bias, heterogeneity, and imprecision (Multimedia Appendix 5).

We ran a sensitivity analysis because some effect sizes seemed to be outliers. Specifically, we excluded a study conducted by Thomas et al [37], given that the anxiety level at baseline was statistically higher (*P*=.01) in the exergame group (mean 8.53, SD 3.62) than in the control group (mean 6.27, SD 3.28). The degree of heterogeneity decreased significantly from 63% to 28% when excluding the results in Thomas et al [37]. The difference in anxiety levels between the groups was statistically significant (*P*=.02; SMD −0.38, 95% CI −0.71 to −0.06), favoring exergames over no intervention (Multimedia Appendix 6). This difference was also clinically important as the overall effect was outside the MCID boundaries (−0.19 to +0.19) and its CI did not cross the *no-effect* line (zero effect). We also performed a sensitivity analysis after excluding another study [36] because anxiety levels at baseline were considerably higher in the control group (mean 6.9, SD 7.4) than in the exergame group (mean 2.7, SD 2.0). However, the degree of heterogeneity and total effect size did not change significantly (Multimedia Appendix 6).

#### Computerized CBT Games

Six studies compared the effect of computerized CBT games with no intervention on anxiety levels [41-46]. While 3 studies did not find a statistically significant difference in anxiety levels between the groups [44-46], the 3 remaining studies showed a statistically significant difference in the anxiety levels between the groups, favoring computerized CBT games over no intervention [41-43].

**Figure 5:**
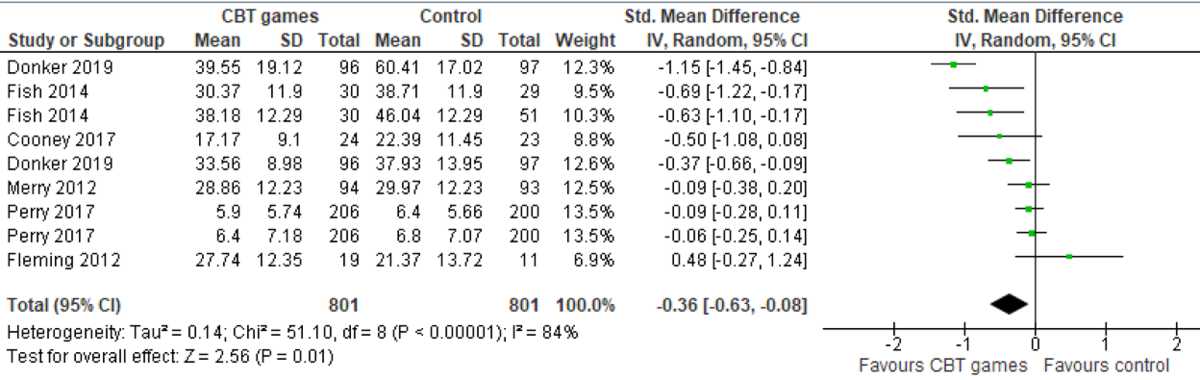
Forest plot of 6 studies (9 comparisons) comparing the effect of CBT games to that of no intervention on the severity of depressive symptoms [41-46]. CBT: cognitive behavioral therapy; Std: standardized.

The results of these 6 studies were included in the meta-analysis. Three of these studies assessed anxiety levels using 2 different measures (Acrophobia Questionnaire [AQ] and Beck Anxiety Inventory [BAI] [42], State-Trait Anxiety Inventory [STAI]–State and STAI-Trait [43], SCAS–Generalized Anxiety Disorder, and SCAS-Social Anxiety [46]). Therefore, we included the results of all these measures in the meta-analysis to form 9 comparisons (Figure 5 [41-46]). The meta-analysis showed a statistically significant difference in the anxiety levels (*P*=.01) between computerized CBT games and control groups, favoring computerized CBT games over no intervention (SMD −0.36, 95% CI −.63 to −0.08). This difference was also clinically important as the overall effect was outside the MCID boundaries (−0.18 to +0.18) and its CI neither crossed the *no-effect* line (zero effect) nor any of the 2 MCID boundaries. For this outcome, MCID boundaries were calculated as −0.5 to +0.5 times the SMD value (−0.36). The statistical heterogeneity of the evidence was considerable (*P*<.001; *I*^2^=84%). The quality of the evidence was very low, as it was downgraded by 5 levels because of a high risk of bias, heterogeneity, and imprecision (Multimedia Appendix 5).

**Figure 6:**
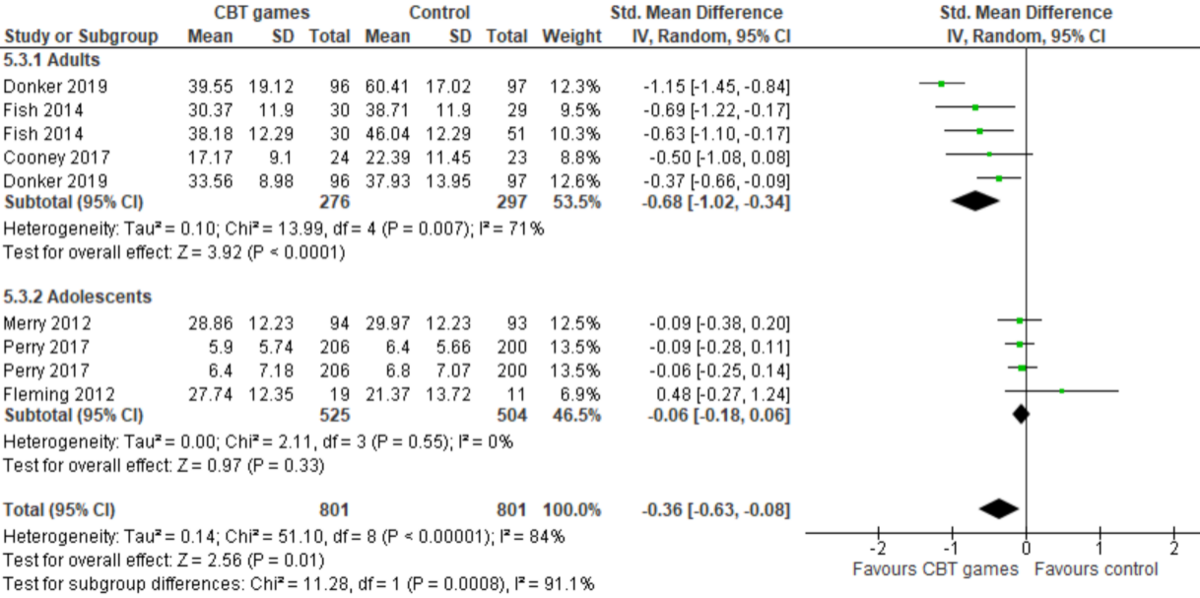
Forest plot of 6 studies (9 comparisons) comparing the effect of CBT games to that of no intervention on the anxiety level among adults and adolescents [41-46]. CBT: cognitive behavioral therapy; Std: standardized.

It is noteworthy that 3 of the 6 studies in the group targeted adults [41-43] while the reminders targeted adolescents [44-46]. Therefore, we conducted a subgroup analysis to assess whether the effect of computerized CBT differs between adults and adolescents. The subgroup analysis showed that the effect of computerized CBT on anxiety was statistically different (*P*<.001) between adults and adolescents (Figure 6 [41-46]). Specifically, while there was no statistically significant difference (*P*=.33) in the anxiety levels between the exergame group and the no intervention group among adolescents (SMD −0.06, 95% CI −0.18 to 0.06), there was a statistically significant difference in the anxiety levels (*P*<.001) between computerized CBT games and control groups among adults (favoring computerized CBT games over no intervention [SMD −0.68, 95% CI −1.02 to −0.34]). The statistically significant difference among adults was also clinically important as the overall effect was outside the MCID boundaries (−0.34 to +0.34) and its CI neither crossed the *no-effect* line (Zero effect) nor any of the 2 MCID boundaries. For this outcome, MCID boundaries were calculated as −0.5 to +0.5 times the SMD value (−0.68). The statistical heterogeneity of the evidence was substantial (*P*=.007; *I*^2^=71%). The quality of the evidence was very low, as it was downgraded by 4 levels because of a high risk of bias and heterogeneity (Multimedia Appendix 5).

Donker et al [42] used two different questionnaires to assess anxiety levels: BAI and AQ-anxiety. The BAI is used to measure general anxiety symptoms while AQ-anxiety measures a specific type of anxiety, which is height-related anxiety [61]. We performed a sensitivity analysis by excluding the AQ-related results reported by Donker et al [61] because all studies in the meta-analysis assessed general anxiety symptoms. The sensitivity analysis showed a significant decrease in the degree of heterogeneity (from 84% to 56%), and the difference in anxiety levels between the groups remained statistically significant (*P*=.01; SMD −0.23, 95% CI −0.41 to −0.05), favoring computerized CBT games over no intervention (Multimedia Appendix 6). This difference remained clinically important as the overall effect was outside the MCID boundaries (−0.12 to +0.12) and its CI did not cross the *no-effect* line (zero effect). We also performed a sensitivity analysis after excluding another study [44] because its sample size (n=30) was relatively small compared with other studies. However, the degree of heterogeneity and total effect size did not change significantly (Multimedia Appendix 6).

#### Biofeedback Games

Biofeedback games were used as interventions in 5 studies [47-51]. Two studies examined the effect of a biofeedback game (MindLight) and conventional CBT on anxiety levels (measured by the SCAS) among children with anxiety [47,48]. Both studies found no statistically significant difference in anxiety levels between the biofeedback game group and the conventional CBT group [47,48].

**Figure 7:**
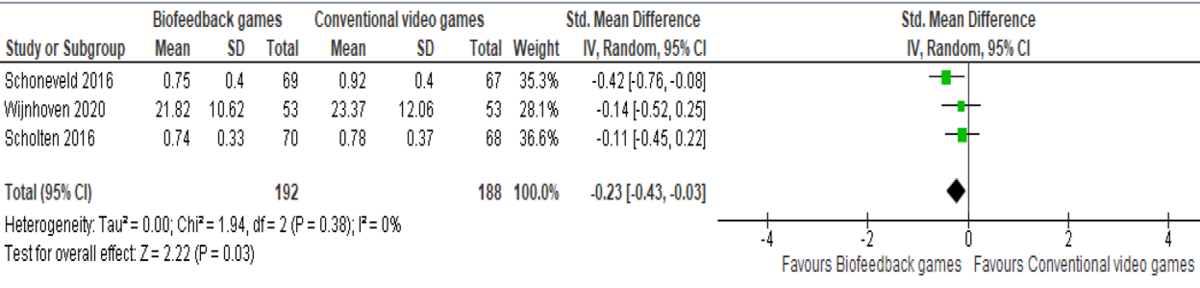
Forest plot of 3 studies comparing the effect of biofeedback games to that of conventional video games on the anxiety level [49-51]. Std: standardized.

The 3 remaining studies examined the effect of biofeedback games and conventional video games on anxiety levels (measured by the SCAS) among children with anxiety [49-51]. While 2 studies did not find a statistically significant difference in anxiety levels between the groups [50,51], the remaining study showed a statistically significant difference in the anxiety level between the groups, favoring biofeedback games over conventional video games [49]. A meta-analysis of the results of these 3 studies demonstrated a statistically significant difference in the anxiety levels (*P*=.03) between the biofeedback game group and conventional CBT group, favoring biofeedback games over conventional video games (SMD −0.23, 95% CI −0.43 to −0.03; Figure 7 [49-51]). This difference was also clinically important as the overall effect was outside the MCID boundaries (−0.115 to +0.115) and its CI neither crossed the *no-effect* line (zero effect) nor any of the 2 MCID boundaries. For this outcome, MCID boundaries were calculated as −0.5 to +0.5 times the SMD value (−0.23). The heterogeneity of the evidence was considered insignificant (*P*=.38; *I*^2^=0%). The quality of the evidence was low, as it was downgraded by 2 levels because of a high risk of bias and imprecision.

#### Attention Distraction Games

Distraction games were used as interventions in 3 studies. Attention distraction games were interventions in 3 studies [52-54]. While 2 studies found a statistically significant effect of attention distraction games [53,54], the remaining study did not [52]. Specifically, Marechal et al [52] compared the effect of attention distraction games with medication (ie, midazolam) on anxiety levels (measured by the Modified Yale Preoperative Anxiety Scale) among children undergoing general anesthesia for minor surgical procedures. No statistically significant difference (*P*=.99) in anxiety levels was detected between the 2 groups [52]. The second study examined the effect of attention distraction games (Angry Birds, Subway Surfers, or Snail Bob), medication (midazolam), and watching an informative cartoon on the anxiety level (measured by the Modified Yale Preoperative Anxiety Scale) among children undergoing adenoidectomy, adenotonsillectomy, or myringotomy [53]. The study showed a statistically significant difference (*P*<.001) in the anxiety level between the groups, favoring the attention distraction games over medication (midazolam) and watching an informative cartoon. In the third study [54], the effect of an attention distraction game (SpaceBurgers) on anxiety levels (measured by Subjective Units of Distress) among children with otolaryngological issues was compared with topical analgesia. The study found a statistically significant difference (*P*<.001) in the anxiety levels between the groups, favoring attention distraction games over topical analgesia [54].

#### Brain Training Games

Brain training games were interventions in 2 studies [55,56]. The first study compared the effect of a brain training game (Tetris) to eye movement desensitization and reprocessing therapy on the levels of trait anxiety (measured by STAI) among patients with posttraumatic stress disorder [55]. The study did not detect any statistically significant difference (*P*=.81) in the level of trait anxiety postintervention [55]. The second study compared the effects of a brain training game (Band Together) and traditional video games on the level of anxiety (measured by STAI) in patients with multiple sclerosis [56]. No statistically significant difference in the levels of state anxiety (*P*=.95) and trait anxiety (*P*=.75) between the 2 groups was detected.

#### Social Skills Training Games

Social skills training games were an intervention in 2 studies [57,58]. The first study investigated the effect of a social skills training game (Adventures) on the anxiety level (measured by the Social Anxiety Scale for Children-Revised) among patients with social skills deficits in comparison with no intervention. The study showed no statistically significant difference (*P*=.10) in anxiety levels between the groups. In the second study, the effect of a social skills training game (Pegasys-Virtual Reality) and social effectiveness therapy on the anxiety level (measured by Social Phobia and Anxiety Inventory for Children) among children with social anxiety were examined. The study demonstrated no statistically significant difference (*P*=.23) in anxiety levels between the groups.

#### Other Types of Serious Games

One study compared the effect of an exposure therapy game (Spider App) to an entertainment video game (Bubble Shooter) on anxiety levels among patients with arachnophobia [59]. No statistically significant difference in anxiety level was detected between the groups postintervention [59].

Litvin et al [60] examined the effect of a psychoeducation game (eQuoo), conventional CBT, and no intervention on anxiety levels among healthy employees. The study did not find any statistically significant difference (*P*=.95) in anxiety levels between the 3 groups [60].

## Discussion

### Principal Findings

This review examined the effectiveness of serious games on anxiety levels, as reported by RCTs. Of the 33 RCTs included in the current review, 20 were included in 4 main meta-analyses. The review found no statistically significant effect of exergames on anxiety levels, though it showed a statistically significant effect of computerized CBT games and biofeedback games on anxiety levels. Owing to the paucity of evidence, no statistical analysis was carried out for other types of serious games included in this review.

Very low–quality evidence from 9 RCTs showed no statistically significant effect of exergames on anxiety levels as compared with conventional exercises. This insignificant effect can be attributed to the fact that exergames are comparable with conventional exercises; therefore, it should not be surprising that comparing the effect of 2 very similar interventions did not produce a significant difference. This indicates that conventional exercises are at least as effective as conventional exercises. Our findings are similar to those of previous reviews [16,62]. Specifically, a meta-analysis of 5 RCTs showed no statistically significant difference (*P*=.81) in anxiety levels between the exergames group and the usual care group (ie, conventional exercises) [16]. Similarly, no statistically significant difference (*P*=.12) in *depression* levels between the exergames group and conventional exercises was found in another meta-analysis of 7 RCTs [62].

Very low–quality evidence from 5 RCTs showed no statistically significant effect when compared with the effects of exergames on anxiety levels as opposed to no intervention. However, a sensitivity analysis of 4 RCTs showed a statistically and clinically significant effect of exergames on anxiety level when compared with no intervention.

This finding is consistent with that of a previous review [16,62]. Specifically, a meta-analysis of 8 studies showed a statistically significant difference (*P*=.004) in *depression* levels between the exergames group and the control group. In contrast, exergames have a statistically and clinically significant effect on *depression* levels when compared with no intervention, according to a meta-analysis of 8 studies [62].

Very low–quality evidence from 6 RCTs demonstrated a statistically and clinically significant effect of computerized CBT games on anxiety levels when compared with no intervention. A subgroup analysis showed that the effect of computerized CBT on anxiety was significantly higher among adults than among adolescents. However, this finding may not be generalizable to older adults as participants in all the 6 studies were, on average, ≤41.3 years. To the best of our knowledge, no previous reviews have examined the effect of computerized CBT games on anxiety, although many reviews have assessed the effect of computerized CBT in general (ie, games are not part of the intervention) [63-66]. However, our findings are in line with a previous review focusing on depression, which found a statistically and clinically significant effect of computerized CBT games on depression level according to a meta-analysis of 6 RCTs.

Low-quality evidence from 3 RCTs showed a statistically and clinically significant effect of biofeedback games on anxiety levels when compared with conventional video games. It is worth mentioning that the studies used biofeedback games specifically for alleviating anxiety and recruited participants with anxiety. The generalizability of this finding may be limited because of the following reasons: (1) participants in the 3 studies were adolescents (10-13.3 years), (2) all studies were conducted in the Netherlands, and (3) there was a small number of studies included in the meta-analysis.

Meta-analyses were not conducted to assess the effect of other types of serious games because of the small number of studies. Individual studies found no statistically significant effect of brain training games, social skills training games, exposure therapy games, and psychoeducation games on anxiety levels. However, other studies have shown contradictory results regarding the effects of attention distraction games on anxiety levels.

### Strengths and Limitations

#### Strengths

This review can be considered more comprehensive than the 2 previous reviews [16,17] because it was not restricted to a certain type of serious games, age group, or comparator, and it searched the main databases in health and information technology fields. This review was conducted according to highly recommended guidelines (ie, PRISMA) and included only RCTs. Therefore, it can be considered a robust and high-quality review.

The risk of publication bias is not a concern in this review because we sought to retrieve as many relevant studies as possible by searching the most popular databases in information technology, health fields, and gray literature databases, conducting backward and forward reference list checking, using a comprehensive search query, and not restricting our search to a certain country, year, setting, population, and comparator.

There is no concern about the risk of selection bias in this review, given that 2 reviewers independently performed the study selection, data extraction, risk of bias assessment, and quality of evidence evaluation with a perfect interrater agreement for all processes. The quality of the evidence was appraised using the GRADE approach to enable the reader to draw more accurate conclusions. When possible, we synthesized data statistically, which improved the power of the studies and increased the estimates of the likely size of the effect of serious games on anxiety.

#### Limitations

This review excluded studies that used serious games delivered on nondigital platforms and those used for other purposes (eg, screening or diagnosis). Therefore, this review cannot comment on the effectiveness of these types of serious games. This review focused on the effectiveness of serious games on anxiety only; thus, we cannot comment on the effectiveness of serious games on other diseases.

Numerous studies were excluded as they were quasi-experiments and written in languages other than English. Therefore, it is likely that we missed relevant studies. We excluded these studies because quasi-experiments have lower internal validity than RCTs [67] and, owing to practical constraints, it was not possible to translate all non-English studies. Participants in most studies did not have anxiety before the intervention; therefore, the effect of serious games could not be significant.

This review meta-analyzed postintervention data rather than follow-up data; thus, this review cannot comment on the long-term effects of serious games on anxiety. Postintervention outcome data were selected given that about half of the included studies (16/33, 48.5%) did not follow-up with participants to measure the outcome data, and the follow-up period in the other half of the studies (17/33, 51.5%) was not consistent between studies.

We used postintervention data for each group to assess the effect size for each meta-analyzed study rather than the pre–post intervention change for each group; therefore, it is likely that the effect size is overestimated or underestimated. We used postintervention outcome data because most studies did not report the SD for pre- or postintervention change for each group, and preintervention outcome data were significantly different between groups in only 2 studies [36,37].

### Research and Practical Implications

#### Research Implications

Although anxiety was one of the measured outcomes in all the included studies, only 6 studies targeted the recruitment of people experiencing anxiety. This may lead to a severe underestimation of the effect of serious games on anxiety levels. This finding is consistent with a similar study that investigated the effects of depression [62]. Similarly, we recommend purposefully recruiting participants who have anxiety and establishing a baseline to objectively assess the effectiveness of serious games in reducing anxiety levels.

We would like to point out that several studies recruited very small samples, with a minimum of only 30 patients. Gaining statistically reliable insights from such small samples can be difficult and may be an additional reason why our meta-analyses provide no conclusive answer to the question of whether serious games can improve or augment traditional anxiety treatment. Thus, we encourage researchers to recruit a sample size that is sufficient to achieve a power of at least 80%.

Most of the included studies were conducted in a clinical setting. Although this could offer a controlled environment to run the studies, it could also introduce stress to the participants because of the nature of such a setting. Conducting more studies in the community and educational settings could present different findings as people usually play games outside of the traditional clinical setting.

The current literature focused mainly on exergames and computerized CBT games, while the effect of other types of serious games was investigated in only a few studies. There are opportunities to enrich the body of evidence on the effectiveness of serious games delivered through other therapeutic modalities such as psychoeducation games, biofeedback games, exposure therapy games, and brain training games.

Although serious games can be used for several purposes and many diseases, we focused on serious games that were used for therapeutic or prevention purposes and anxiety only. Researchers should conduct systematic reviews to assess the effectiveness of serious games used for other purposes (eg, monitoring, screening, and diagnosing) and for other diseases.

In only 2 studies, the overall risk of bias was low given that most studies had issues in the randomization process, measurement of the outcome, and selection of the reported result. Outcome data were missing from several studies; therefore, they were not included in the meta-analyses. Accordingly, researchers should avoid the abovementioned biases by conducting and reporting RCTs according to recommended guidelines or tools (eg, RoB 2 [25]).

Finally, most of the included studies were conducted in high-income countries, which, in turn, can limit the generalizability of our findings to low-income nations. There is a need to conduct more studies in low-income countries, especially given the varying nature of their cultures, socioeconomic conditions, and sources of stress and anxiety (eg, overpopulated cities, poor socioeconomic areas, and refugee camps). Furthermore, more studies are needed to determine any variance in the effectiveness of serious games that are designed specifically to reduce and alleviate anxiety levels intergenerationally.

#### Practical Implications

This review showed that exergames are as effective as conventional exercises in alleviating anxiety and that computerized CBT games and biofeedback games are more effective than no intervention and conventional video games, respectively. However, health professionals and decision-makers should be careful when interpreting these findings for the following reasons: the quality of meta-analyzed evidence ranged from very low to low, the overall risk of bias was high in most of the included studies, the heterogeneity of the evidence was high in the 3 meta-analyses, participants in most studies did not have anxiety, and many studies did not use serious games that were designed specifically to alleviate anxiety. Accordingly, psychologists and psychiatrists should consider offering serious games as complementary and not a substitute for existing interventions until further, more robust evidence is available.

Although anxiety can be alleviated by many nonpharmacological interventions, there are no or few serious games that deliver nonpharmacological interventions other than exercises and CBT in this review. This may be attributed to the lack of such serious games in real life. Therefore, developers should consider developing serious games that deliver nonpharmacological interventions such as breathing techniques, mindfulness training, problem-solving, attention distraction, biofeedback, psychoeducation, relaxation-based exercises, and rational emotive behavioral therapy.

Only a handful (n=7) of studies used mobile devices (smartphones and tablets) as the platform for their intervention. Mobile devices are particularly appealing because they are cheaper than computers and more pervasive than gaming consoles. Moreover, mobile devices are more accessible than computers and gaming consoles; it is estimated that there are approximately 15 billion mobile devices and more than 7.1 mobile users worldwide in 2021 [68]. This could present a lucrative opportunity for app and game developers to develop serious games that target anxiety and can be played via mobile devices.

Few studies have been conducted in developing countries, and this may be attributed to the lack of serious games in these countries. Given that there is a greater shortage of mental health professionals in developing countries than in developed countries (0.1 per 1 million people [69] versus 90 per 1 million people [70]), it is likely that individuals in developing countries are more in need of serious games than those in developed countries. Therefore, more serious games should be developed to alleviate anxiety among people in developing countries.

We would like to point out that a significant portion of the studies (n=12) investigated intervention methods using now-discontinued platforms: Wii (n=8, end of life in 2017), Kinect (n=5, end of life in 2017), and Nintendo DS (n=1, end of life in 2014). Only in one case, other platforms will readily fill the gap in only one case (using Tetris [52]). For interventions using Microsoft’s Kinect sensor, computer vision–based pose estimation on mobile phones or desktop PCs could fill the gap but will result in a different setup. Finally, some of the included studies using Wiimote (Wii Remote) and none of the more specialized Wii input devices could be recreated using newer Nintendo controllers. These considerations raise a few questions of practical importance: (1) How well can studies relying on legacy and specialized hardware be reproduced? (2) How useful are interventions that rely on platforms designed to undergo comparatively short life cycles? (3) Are off-the-shelf video games (purpose-shifted games) adequate intervention tools?

We believe that some of the included studies relying on legacy hardware could probably be salvaged, following the comments outlined above, but caution should be taken to fall victim to the novelty effect of emerging game controllers and proprietary input devices. The video game industry evolves quickly and is known to experiment with novel technology to spirit gamers away from competitors. Consequently, purpose-shifted games are not only very prone to depreciate quickly, but the same is true for the platforms they were designed for. We believe that researchers in this space should best assume the role of game designers, who focus on the game mechanics and purpose. In the second step, researchers are probably best advised to seek the help of a professional software development company to bring out the product in a timely fashion.

In addition, although we cannot rule out that off-the-shelf games that have undergone, first, a purpose-shift to become a serious game and yet another one to become part of a therapy (eg, Tetris) have a measurable effect, we also have little reason to assume that they do. It seems tempting to explain the effects of serious games on anxiety by their distractive nature, but studies do not agree with this question.

There is also an urgent need for an inclusive approach when developing these apps and games to include professionals from the gaming industry as well as mental health experts. Technologists and developers are usually very aware of the aforementioned concerns but need medical professionals to avoid falling prey to the temptation of purpose-shifting existing games or designing games for goals that are different from anxiety relief.

### Conclusions

Evidence from this study suggests that serious games have the potential to reduce anxiety levels. Specifically, exergames can be as effective as conventional exercises in alleviating anxiety; computerized CBT games and exergames can be more effective than no intervention, and biofeedback games can be more effective than conventional video games. However, definitive conclusions regarding the effectiveness of serious games in reducing anxiety remain inconclusive, mainly because of the high risk of bias in the individual studies included, the low quality of meta-analyzed evidence, the low number of studies included in some meta-analyses, participants without anxiety in most studies, and using purpose-shifted serious games in most studies. Until further, more robust evidence is available, serious games should be deemed as complementary to existing interventions and not as a substitute for them. To obtain adequate and robust evidence, researchers should use serious games specifically designed to alleviate depression and deliver other therapeutic modalities, recruit patients with anxiety, and minimize the risk of bias by recommended guidelines for conducting and reporting RCTs (eg, RoB 2).

## Appendix

Multimedia Appendix 1 PRISMA (Preferred Reporting Items for Systematic Reviews and Meta-Analyses) checklist.

Multimedia Appendix 2 Search strategy.

Multimedia Appendix 3 Data extraction form.

Multimedia Appendix 4 Reviewers’ judgments about each risk of bias domain for each included study.

Multimedia Appendix 5 Grading of Recommendations Assessment, Development, and Evaluation profile for the comparison of serious games to control or conventional exercises for anxiety.

Multimedia Appendix 6 Sensitivity analyses.

## Abbreviations

AQ: Acrophobia Questionnaire
BAI: Beck Anxiety Inventory
CBT: cognitive behavioral therapy
GRADE: Grading of Recommendations Assessment, Development, and Evaluation
MCID: minimal clinically important difference
PRISMA: Preferred Reporting Items for Systematic Reviews and Meta-Analyses
PROSPERO: International Prospective Register of Systematic Reviews
RCT: randomized controlled trial
RoB 2: Risk-of-Bias 2
SCAS: Spence Children’s Anxiety Scale
SMD: standardized mean difference
STAI: State-Trait Anxiety Inventory
