# Supplementary material for: The Effectiveness of Serious Games in Alleviating Anxiety: Systematic Review and Meta-analysis
Source: JMIR Serious Games. 2022 Feb 14;10(1):e29137. doi: 10.2196/29137 (PMC8887639; doi:10.2196/29137)
Supplement: Multimedia Appendix 2 [file games_v10i1e29137_app2.docx]

**Appendix 2 Search strategy**

Database(s): **Ovid MEDLINE(R) ALL**1946 to 29 June, 2021
Search Strategy:

| **#** | **Searches** | **Results** |
| --- | --- | --- |
| 1 | exp Anxiety Disorders/ | 81633 |
| 2 | "anxiety".tw. | 39523 |
| 3 | anxious.tw. | 17953 |
| 4 | 1 or 2 or 3 | 101986 |
| 5 | exp Video Games/ | 5769 |
| 6 | "serious gam*".tw. | 772 |
| 7 | "game-based".tw. | 662 |
| 8 | "videogam*".tw. | 775 |
| 9 | "video game*".tw. | 3496 |
| 10 | gamification.tw. | 603 |
| 11 | gamified.tw. | 306 |
| 12 | exergam*.tw. | 706 |
| 13 | "Applied game*".tw. | 20 |
| 14 | virtual reality game*.tw. | 121 |
| 15 | Virtual reality-based game*.tw. | 3 |
| 16 | augmented reality game*.tw. | 29 |
| 17 | augmented reality-based game*.tw. | 0 |
| 18 | 5 or 6 or 7 or 8 or 9 or 10 or 11 or 12 or 13 or 14 or 15 or 16 or 17 | 9499 |
| 19 | exp Randomized Controlled Trial/ | 522353 |
| 20 | "Randomized Controlled Trial*".tw. | 146314 |
| 21 | "Randomised Controlled Trial*".tw. | 46488 |
| 22 | "Randomized Control Trial*".tw. | 7496 |
| 23 | "Randomised Control Trial*".tw. | 1965 |
| 24 | experiment*.tw. | 2119098 |
| 25 | "clinical trial*".tw. | 389950 |
| 26 | 19 or 20 or 21 or 22 or 23 or 24 or 25 | 3012512 |
| 27 | 4 AND 18 AND 26 | 283 |
| 28 | limit 110 to english language | 256 |

**CINHAL (EBSCO)**

| **#** | **Query** | **Results** |
| --- | --- | --- |
| S1 | ((MH "anxiety disorder*") OR (AB anxiety) OR (TI anxiety) OR (AB anxious) OR (TI anxious)) | 95,131 |
| S2 | MH serious games | 489 |
| S3 | TI "serious gam*" OR AB "serious gam*" | 376 |
| S4 | TI "game-based" OR AB "game-based" | 411 |
| S5 | TI "videogame*" OR AB "videogame*" | 307 |
| S6 | TI "video game*" OR AB "video game*" | 2,170 |
| S7 | TI "virtual reality game*" OR AB "virtual reality game*" | 63 |
| S8 | TI "virtual reality-based game*" OR AU "virtual reality-based game*" | 0 |
| S9 | TI "Augmented Reality-based game" OR AB "Augmented Reality-based game" | 0 |
| S10 | TI "Augmented Reality game*" OR AB "Augmented Reality game*" | 21 |
| S11 | TI "gamification" OR AB "gamification" | 355 |
| S12 | TI gamified OR AB gamified | 159 |
| S13 | TI exergam* OR AB exergam* | 362 |
| S14 | TI "Applied game*" OR AB "Applied game*" | 8 |
| S15 | S2 OR S3 OR S4 OR S5 OR S6 OR S7 OR S8 OR S9 OR S10 OR S11 OR S12 OR S13 OR S14 | 4,825 |
| S16 | MH Randomized Controlled Trials | 112,798 |
| S17 | TI Randomized Controlled Trial* OR AB Randomized Controlled Trial* | 93,812 |
| S18 | TI Randomised Controlled Trials OR AB Randomised Controlled Trials | 21,159 |
| S19 | TI "Randomised Control Trial*" OR AB "Randomised Control Trial* | 1,383 |
| S20 | TI "Randomized Control Trial*" OR AB "Randomized Control Trial*" | 3,607 |
| S21 | TI Experiment* OR AB Experiment* | 140,991 |
| S22 | TI "Clinical trial*" OR AB "Clinical trial*" | 112,633 |
| S23 | S16 OR S17 OR S18 OR S19 OR S20 OR S21 OR S22 | 398,482 |
| S24 | S1 AND S15 AND S23 | 65 |

**APA PsycInfo (EBSCO)**

| **#** | **Query** | **Results** |
| --- | --- | --- |
| S1 | ((MH "anxiety disorder*") OR (AB anxiety) OR (TI anxiety) OR (AB anxious) OR (TI anxious)) | 142,323 |
| S2 | MH serious games | 620 |
| S3 | TI "serious gam*" OR AB "serious gam*" | 410 |
| S4 | TI "game-based" OR AB "game-based" | 503 |
| S5 | TI "videogame*" OR AB "videogame*" | 355 |
| S6 | TI "video game*" OR AB "video game*" | 2,301 |
| S7 | TI "virtual reality game*" OR AB "virtual reality game*" | 72 |
| S8 | TI "virtual reality-based game*" OR AU "virtual reality-based game*" | 0 |
| S9 | TI "Augmented Reality-based game" OR AB "Augmented Reality-based game" | 0 |
| S10 | TI "Augmented Reality game*" OR AB "Augmented Reality game*" | 42 |
| S11 | TI "gamification" OR AB "gamification" | 366 |
| S12 | TI gamified OR AB gamified | 173 |
| S13 | TI exergam* OR AB exergam* | 375 |
| S14 | TI "Applied game*" OR AB "Applied game*" | 15 |
| S15 | S2 OR S3 OR S4 OR S5 OR S6 OR S7 OR S8 OR S9 OR S10 OR S11 OR S12 OR S13 OR S14 | 5,207 |
| S16 | MH Randomized Controlled Trials | 112,772 |
| S17 | TI Randomized Controlled Trial* OR AB Randomized Controlled Trial* | 93,777 |
| S18 | TI Randomised Controlled Trials OR AB Randomised Controlled Trials | 21,250 |
| S19 | TI "Randomised Control Trial*" OR AB "Randomised Control Trial* | 1,451 |
| S20 | TI "Randomized Control Trial*" OR AB "Randomized Control Trial*" | 3,607 |
| S21 | TI Experiment* OR AB Experiment* | 142,203 |
| S22 | TI "Clinical trial*" OR AB "Clinical trial*" | 112,733 |
| S23 | S16 OR S17 OR S18 OR S19 OR S20 OR S21 OR S22 | 496,956 |
| S24 | S1 AND S15 AND S23 | 218 |
| S25 | S1 AND S15 AND S23; Narrow by Language: - english | 167 |

| **Database** | **Query** | **Results** |
| --- | --- | --- |
| **Scopus** | ( TITLE-ABS-KEY (anxiety OR anxious ) ) AND ( TITLE-ABS-KEY ( "serious gam*" OR "game-based" OR "videogame*" OR "video game*" OR "virtual reality game*" OR "virtual reality-based game*" OR "Augmented Reality-based game" OR "Augmented Reality game*" OR "gamification" OR gamified OR exergam* OR "Applied game*" ) ) AND ( TITLE-ABS-KEY ( "randomized controlled trial*" OR "randomised controlled trial*" OR "randomized control trial*" OR "randomised control trial*" OR "clinical trial*" OR experiment* ) ) AND ( LIMIT-TO ( DOCTYPE , "ar" ) OR LIMIT-TO ( DOCTYPE , "cp" ) ) AND ( LIMIT-TO ( EXACTKEYWORD , "Human" ) ) AND ( LIMIT-TO ( LANGUAGE , "English" ) ) | 324 |
| **IEEE Xplore** | ((((Abstract:control trial* OR controlled trial*))) AND ((Abstract:anxiety OR anxious) AND ((Abstract:"serious gam*" OR "game-based" OR "video game*" OR "gamification" OR exergam*)) | 0 |
| **ACM Digital library** | [[Abstract: anxiety] OR [Abstract: anxious]] AND [[Abstract: "serious gam*"] OR [Abstract: "game-based"] OR [Abstract: "videogame*"] OR [Abstract: "video game*"] OR [Abstract: "virtual reality game*"] OR [Abstract: "virtual reality-based game*"] OR [Abstract: "augmented reality-based game*"] OR [Abstract: "augmented reality game*"] OR [Abstract: "gamification"] OR [Abstract: gamified] OR [Abstract: exergam*] OR [Abstract: "applied game*"]] AND [[All: "randomized controlled trial*"] OR [All: "randomised controlled trial*"] OR [All: "randomized control trial*"] OR [All: "randomised control trial*"] OR [All: "clinical trial*"] OR [All: experiment*]] | 23 |
| **Google Scholar** | (anxiety OR anxious) AND ("serious gam*" OR "game-based" OR exergam* OR gamify* OR "video game*" OR "virtual reality game*") AND ("controlled trial*" OR "control trial*") | 100 |
