# Supplementary material for: The Effectiveness of Serious Games in Alleviating Anxiety: Systematic Review and Meta-analysis
Source: JMIR Serious Games. 2022 Feb 14;10(1):e29137. doi: 10.2196/29137 (PMC8887639; doi:10.2196/29137)
Supplement: Multimedia Appendix 4 [file games_v10i1e29137_app4.docx]

**Appendix 4: Reviewers’ judgements about each “risk of bias” domain for each included study**
