# Supplementary material for: The Effectiveness of Serious Games in Alleviating Anxiety: Systematic Review and Meta-analysis
Source: JMIR Serious Games. 2022 Feb 14;10(1):e29137. doi: 10.2196/29137 (PMC8887639; doi:10.2196/29137)
Supplement: Multimedia Appendix 5 [file games_v10i1e29137_app5.docx]

**Appendix 5: GRADE Profile for comparison of Serious games to control or conventional exercises for Anxiety**

| **Certainty assessment** | | | | | | | **Summary of findings** | | | | |
| --- | --- | --- | --- | --- | --- | --- | --- | --- | --- | --- | --- |
| **Participants  (studies) Follow up** | **Risk of bias** | **Inconsistency** | **Indirectness** | **Imprecision** | **Publication bias** | **Overall certainty of evidence** | **Study event rates (%)** | | **Relative effect (95% CI)** | **Anticipated absolute effects** | |
|  |  |  |  |  |  |  | **With placebo** | **With Serious games for Anxiety** |  | **Risk with placebo** | **Risk difference with Serious games for Anxiety** |
| **Exergames vs. Conventional exercises** | | | | | | | | | | | |
| 375 (9 RCTs) | very serious ^a^ | very serious ^b^ | not serious | very serious ^c,d^ | none | ⨁◯◯◯ VERY LOW | 189 | 186 | - | - | SMD **0.07 lower** (0.45 lower to 0.3 higher) |
| **Exergames vs. Control** | | | | | | | | | | | |
| 281 (5 RCTs) | very serious ^e^ | very serious ^f^ | not serious | very serious ^c,g^ | none | ⨁◯◯◯ VERY LOW | 141 | 140 | - | - | SMD **0.23 lower** (0.63 lower to 0.18 higher) |
| **CBT games vs. Control** | | | | | | | | | | | |
| 1602 (6 RCTs) | very serious ^h^ | very serious ^i^ | not serious | serious ^j,k^ | none | ⨁◯◯◯ VERY LOW | 801 | 801 | - | - | SMD **0.36 lower** (0.63 lower to 0.08 lower) |
| **Biofeedback games vs. Conventional video games** | | | | | | | | | | | |
| 380 (3 RCTs) | serious ^l^ | not serious | not serious | serious ^g,k^ | none | ⨁⨁◯◯ LOW | 188 | 192 | - | - | SMD **0.23 lower** (0.43 lower to 0.03 lower) |

**CI:** Confidence interval; **SMD:** Standardised mean difference

#### Explanations

a. Evidence was downgraded by 2 levels because the overall risk of bias was rated as high in five studies and there were some concerns in three studies due to issues mainly in the randomization process and selection of the reported results.

b. Evidence was downgraded by 2 levels as P=0.002 and I^2^ =67%, indicating high heterogeneity.

c. Evidence was downgraded by 2 levels because 95% CI crosses the 2 MID boundaries for this outcome.

d. MID for this outcome, calculated as ± 0.5 times the standardized mean difference (SMD), is ± 0.035

e. Evidence was downgraded by 2 levels because the overall risk of bias was rated as high in four studies due to issues mainly in the randomization process, measurement of the outcome, and selection of the reported results.

f. Evidence was downgraded by 2 levels as P=0.03 and I^2^=63%, indicating high heterogeneity.

g. MID for this outcome, calculated as ± 0.5 times the standardized mean difference (SMD), is ± 0.115

h. Evidence was downgraded by 2 levels because the overall risk of bias was rated as high in four studies and there were some concerns in two studies due to issues mainly in the selection of the reported results and measurement of the outcome.

i. Evidence was downgraded by 2 levels as P<0.001 and I^2^=84%, indicating high heterogeneity.

j. MID for this outcome, calculated as ± 0.5 times the standardized mean difference (SMD), is ± 0.18

k. Evidence was downgraded by 1 level because 95% CI crosses one of MID boundaries for this outcome.

l. Evidence was downgraded by 1 level because the overall risk of bias was rated as high in one study and there were some concerns in one study due to issues mainly in the measurement of the outcome.
