# Supplementary material for: The Effectiveness of Serious Games in Alleviating Anxiety: Systematic Review and Meta-analysis
Source: JMIR Serious Games. 2022 Feb 14;10(1):e29137. doi: 10.2196/29137 (PMC8887639; doi:10.2196/29137)
Supplement: Multimedia Appendix 6 [file games_v10i1e29137_app6.docx]

**Appendix 6: Sensitivity analyses**

**
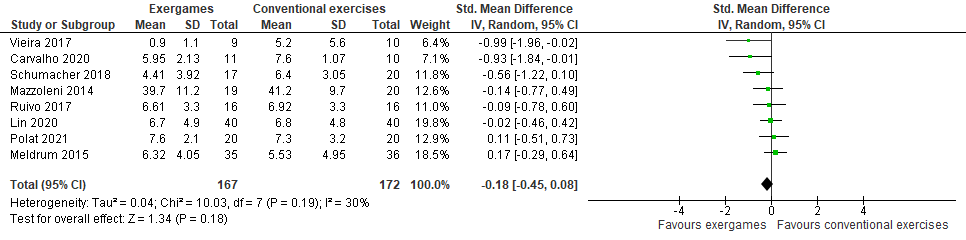
Exergames versus conventional exercises (excluding Adomaviciene et al.)**

**Exergames versus conventional exercises (excluding Adomaviciene et al. and Vieira et al.)**

**
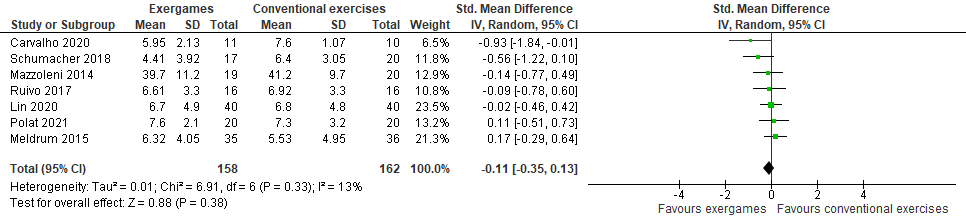
**

**Exergames versus no intervention (excluding Thomas et al.)**

**
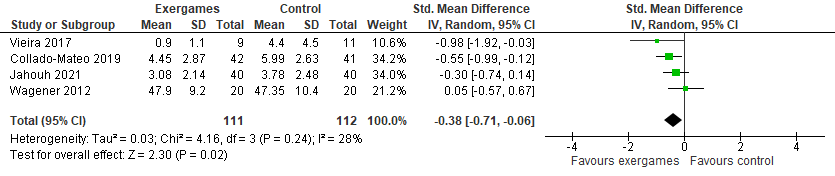
**

**
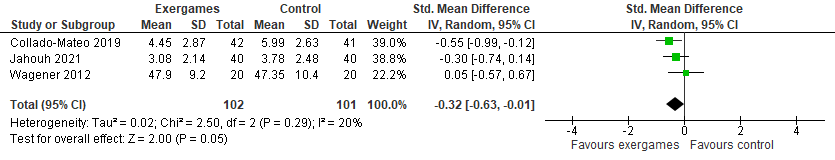
Exergames versus no intervention (excluding Thomas et al. and Vieira et al.)**

**
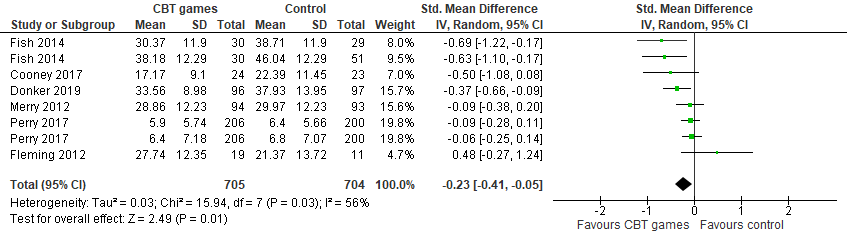
Computerized CBT games versus no intervention (excluding Donker et al.)**

**
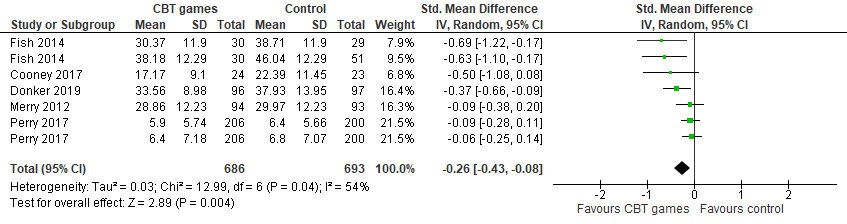
Computerized CBT games versus no intervention (excluding Donker et al. and Fleming et al.)**
